# Catalytic liquefaction of municipal sewage sludge over transition metal catalysts in ethanol-water co-solvent

**DOI:** 10.1016/j.biortech.2017.09.205

**Published:** 2018-02

**Authors:** Wenjia Wang, Qi Yu, Han Meng, Wei Han, Jie Li, Jinglai Zhang

**Affiliations:** a School of Environment and Natural Resource, Renmin University of China, Beijing 100872, PR China; b Maoming R&P Petrochemical Engineering Co., Ltd, Maoming, Guangdong 525011, PR China

**Keywords:** Sludge, Catalytic liquefaction, Ethanol-water, Biocrude

## Abstract

Catalytic liquefaction of (Municipal sewage sludge) MSS over transition metal catalysts in ethanol-water co-solvent (EWCS) was investigated. The effect of operating parameters like temperature, holding time, and ethanol-water ratio was discussed. CuSO_4_ was selected as the most efficient catalyst. The highest biocrude yield (47.45%) and liquefaction conversion (97.74%) was both obtained at the same conditions following: a reaction temperature of 270 °C, a holding time of 30 min, an ethanol-water ratio of 1:1, and with CuSO_4_ as the catalyst. Optimized operating conditions reduced Sulfur and Nitrogen content in biocrude by 55.0% and 14.6%, respectively. The obtained biocrude samples were analyzed and characterized by thermogravimetric analysis (TGA) and gas chromatography-mass spectroscopy (GC-MS), which suggested that adding CuSO_4_ increased the light-oil like content in biocrude and more than 60% of compounds in biocrude were esters. This process demonstrates the effectiveness of catalytic liquefaction of MSS in EWCS over CuSO_4_ catalyst.

## Highlights

• Municipal sewage sludge was 97.74% liquefied with CuSO_4_ in ethanol-water co-solvent.
• CuSO_4_ reduced the content of N and S in biocrude by 14.6% and 55.0%, respectively.
• Appropriate conditions concentrated more C and H in biocrude.
• More light-oil contents and esters were in biocrude with CuSO_4_ catalyst.

## 1 Introduction

With the fast development of society and economy in the past decades, the amount of municipal sewage sludge (MSS) discharge increases rapidly in China. At present, there are over 3800 wastewater treatment plants totally in China, discharging more than 6 million tons of dry sludge per year (Zhang et al., 2016b). This huge amount of MSS causes serious pollution and is urgent to be disposed. Nowadays, there are three major MSS treatment technologies: land filling, land application, and incineration. However, all of these methods require extra energy inputs and inevitably cause secondary pollution (Babel and del Mundo Dacera, 2006). Meanwhile, as the byproduct of municipal wastewater treatment, the high-moisture-content MSS, which is rich in nitrogen, phosphorus, organic matter and micronutrients, is a potential source of bioenergy(Suzuki et al., 1988). The utilization of MSS has aroused interests recently, and various methods were applied by researchers (Caporgno et al., 2016, Choi et al., 2017, He et al., 2014, Papa et al., 2017, Thomsen et al., 2017). As a fast-developing thermochemical conversion method, direct liquefaction is a promising technology to handle the treatment of MSS and produce liquid fuel biocrude simultaneously (Fan et al., 2016, Lemoine et al., 2013).

Direct liquefaction is a thermochemical conversion method allowing to performed at low temperature (200–400 °C), with a holding time (0.25–2 h), and relatively operating pressure (5–20 MPa) (Leng et al., 2015). This thermochemical process can convert biomass into oily compounds in water or other suitable solvents. The solvent extremely influenced the reaction operating condition, the product yield, and biocrude composition (Liu and Zhang, 2008, Zhang et al., 2010). Water, the free and natural green solvent, was often used as the reaction medium in biomass liquefaction. However, the higher critical temperature (374 °C) and critical pressure (22.1 MPa) of water meant challenging operating conditions and extra energy input (Huber et al., 2006). As a cheap and easily available industrial product, ethanol was introduced into the liquefaction process as the reaction medium because of several advantages (Chumpoo and Prasassarakich, 2010): First, the critical temperature and pressure of ethanol are much lower than that of water. Second, as an active hydrogen donator during liquefaction process, ethanol is a suitable polar protic solvent which favors the SN_1_ (unimolecular nucleophilic substitution) reaction in the liquefaction system (Akhtar and Amin, 2011). Third, ethanol can react with acidic compounds by esterification reaction and provide the biodiesel-like biocrude product. However, ethanol is mutually soluble with the target product biocrude, which makes the separation of biocrude and ethanol solvent extremely difficult. Thus, ethanol-water co-solvent (EWCS) would be an efficient reaction medium during the liquefaction process of biomass (Chen et al., 2012, Yuan et al., 2007).

To the best of our knowledge, there is only limited information available on catalytic liquefaction of biomass, especially for treatment of MSS (Prajitno et al., 2017). Moreover, because of various metal elements in industrial sewage sludge (Huang and Yuan, 2016), to use metal salt catalysts in liquefaction of MSS would provide a new method for reusing the metal pollutants and bioenergy simultaneously for the treatment of industrial sewage sludge like electroplating sludge and metallurgical sludge in the future. Thus, it was felt to be of considerable value to fill this gap.

The objectives of this work were to investigate the thermochemical liquefaction of MSS with ethanol and water as the co-solvent over different transition metal salt catalysts, and to explore the effect of reaction temperature, holding time, ethanol/water ratio (E/W) on liquefaction conversion and biocrude yield. The elemental and chemical compositions of the aim product biocrude were analyzed. Meanwhile, the boiling point distributions of biocrude were comparatively evaluated by the themogravimetric analysis (TGA).

## 2 Material and method

### 2.1 Materials

In this paper, the MSS was collected from the secondary sedimentation tanks of Qinghe wastewater treatment plant (WWTP) located in Beijing, China. The MSS samples were dried at 105 °C for 12 h to remove moisture, then ground and sieved until particle size was smaller than 80 mesh. Major elements in the ashes of MSS were analyzed by X-ray fluorescence spectroscopy (XRF, PANalytical, Advanced-Axios, Netherlands), while trace elements were determined by inductively coupled plasma-optical emission spectroscopy (ICP-OES, Perkinelmer, Optima 2000DV, USA). Table 1 lists the properties of the dried MSS. All chemical reagents throughout the experiments were commercially available with analytical grade and used without further purification. All the water used in this paper was deionized water. Critical temperatures and pressures for using ethanol-water solvents with different ethanol-water ratios from literature (Yuan et al., 2007) are given in Table 2.

**Table 1:** Proximate and ultimate analysis of sewage sludge sample.

Mar. (wt%)		Proximate analysis (d, wt%)												Ultimate analysis (daf, wt%)											
		A			Lipid			Protein			Sugar			C			H	O^a^			N			S	
84.52		23.01			8.01			37.84			31.14			46.68			6.85	37.60			8.05			0.81	

Major elements weight content in ash (wt%)																									

Al_2_O_3_	CaO			Cl			Fe_2_O_3_		K_2_O			MgO			Na_2_O		P_2_O_5_		SiO_2_			SO_3_			TiO_2_
16.27	16.54			4.28			7.37		3.88			3.87			8.29		12.87		12.69			13.66			0.27

Trace elements weight content in ash (μg/g)																									

Cd			Co			Cr				Cu			Mn			Ni				V			Zn		
8			22			314				1435			273			126				63			1368		

M: moisture; A: ash; ar: as received basis; d: dry basis; daf: dry and ash-free basis.

a Calculated by difference.

**Table 2:** The critical temperature and pressure (Tc, Pc) for used solvents with different ethanol-water ratio (°C, MPa).

Ethanol-water Ratio	Tc	Pc
0:10	374.15	22.10
1:9	362.92	19.89
3:7	339.38	15.56
5:5	313.97	11.67
6:4	299.49	10.04
8:2	271.79	7.82
10:0	243.25	6.30

### 2.2 Apparatus and procedure of MSS liquefaction

Direct liquefaction process was conducted in a batch stainless 316 steel autoclave reactor with 600 mL capacity from Weihai Chemical Machinery CO., Ltd. (Shandong, China). The reactor was designed to operate at a maximum temperature of 360 °C and a maximum pressure of 30 MPa. The autoclave was heated with an external electrical furnace, and the temperature was measured with a thermocouple.

In a typical experiment, 60 g of dried MSS, 240 mL of ethanol-water solvent and 6 g of catalyst were fed into the reactor and then purged with pure N_2_ three times to replace the air. After that, the reactor was sealed and heated up to desired reaction temperature. During the liquefaction process, the reactants were agitated vertically using a magnetically coupled mechanical stirrer (95 rpm). The holding time was counted from the moment that the reactor reached the set temperature.

### 2.3 Product separation procedure

After liquefaction process, the reactor was cooled down to 30 °C with recirculating cooling water systems and then depressurized. The gas product was vented without further analyzed as the very little gas product. Subsequently, the reactor was washed three times with dichloromethane (DCM). The mixture, which consisted of water, ethanol, biocrude, solid matters, aqueous product, and DCM, was filtered. The filtered cake was washed with DCM to remove other product and solvent, then dried in a vacuum drying chamber at 105 °C for 12 h to constant weight and designated as solid residue (SR). The SR samples were ashed in an air atmosphere at 800 °C and weighted. The difference between the weight of SR and the ashes was calculated to be the weight of remained-solid-phase organic matters, which were defined as solid organic residues (OSR). The remaining liquid was further separated into DCM phase and EWCS phase with a separating funnel. DCM was removed in a rotary evaporator under reduced pressure at 70 °C. Ultimately, the dark ropy DCM-soluble liquid was weighed and designated as biocrude.

### 2.4 Analytic methods for products

Thermogravimetric analysis (TGA) of biocrude was performed with an integrated thermal gravimetric analyzer (TG-600, Japan) in a nitrogen atmosphere (purity of 99.99%). Biocrude samples were heated from room temperature (25 °C) to 800 °C with a constant heated rate of 10 °C/min and a gas flow rate of 100 mL/min. The boiling range of the biocrude was based on literature (Robin et al., 2017). The elemental compositions of C, H, N, and S in the biocrude samples were analyzed with a CE-400 elemental analyzer (EAI, USA). The element content of O was calculated by difference. The chemical compositions of biocrude samples were analyzed by a gas chromatography-mass spectroscopy (GC–MS). The QP2010 Plus spectrometer (Shimadzu, Japan) was equipped with RTX-5MS capillary column (30 m × 0.25 mm × 0.25 μm). Helium was adopted as the carrier gas with a flow rate of 1.0 mL/min. The column temperature was programmed from 50 to 300 °C at a rate of 10 °C/min and was kept at 300 °C for 10 min. The inlet temperature was set to 300 °C. The ionizing voltage of mass spectrometer was 70 eV, and the mass range was from 20 to 650 aum.

### 2.5 Calculation methods

Liquefaction conversion of MSS, which was to evaluate how much organic matters in MSS were transferred into non-solid phase matters, was calculated by Eq. (1). Biocrude yield was calculated by Eq. (2), and element enrichment ratio (ECR) was calculated by Eq. (3).

(1) $$\text{Liquefaction Conversion}(\text{wt}\%)=\left(1-\frac{{\text{m}}_{\text{OSR}}}{{\text{m}}_{\text{OM}}}\right)\times 100\%$$

(2) $$\text{Biocrude yield}(\text{wt}\%)=\frac{{\text{m}}_{\text{B}}}{{\text{m}}_{\text{OM}}}\times 100\%$$

(3) $$\text{ECR}(\%)=\frac{{\text{E}}_{\text{B}}\times {\text{m}}_{\text{B}}}{{\text{E}}_{\text{OM}}\times {\text{m}}_{\text{OM}}}\times 100\%$$

where m_OSR_, the weight of organic solid reside (g); m_OM_, the weight of organic matters in MSS (g); m_B_, the weight of biocrude (g); E_B_, the element content in biocrude; E_OM_, the element content in organic matter in MSS (%). The weight of organic matters was calculated by the difference between the weight of dry MSS and the ashes in dry MSS. The Higher heating values (HHVs) of samples were calculated as described literature (Zeb et al., 2017). All the experimental errors for liquefaction conversion and biocrude yields are lower than 5% by repeating experiments at least three runs at the same conditions.

## 3 Results and discussion

### 3.1 Effect of catalyst on liquefaction of MSS

#### 3.1.1 Liquefaction results over different transition metal catalyst

**Fig. 1:**
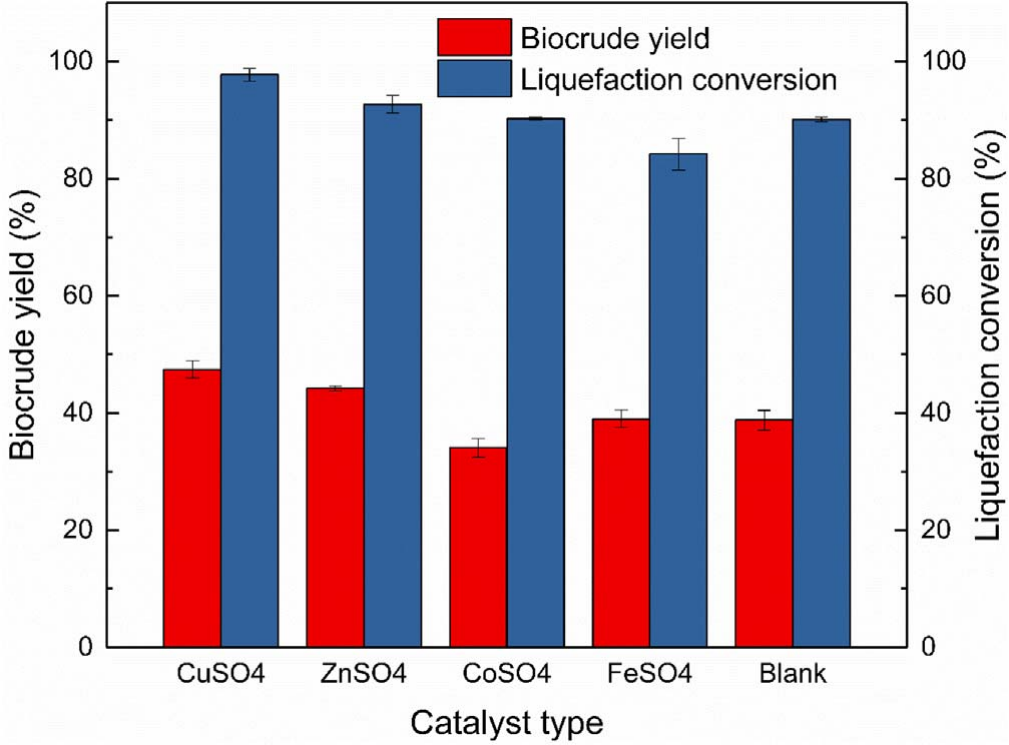
Effect of catalyst types on biocrude yield and liquefaction conversion from catalytic liquefaction of MSS.

The effects of different catalysts (CuSO_4_, CoSO_4_, FeSO_4,_ and ZnSO_4_) on the catalytic liquefaction of MSS are shown in Fig. 1 and Table 3. Further comparison with the process without catalysts is also presented. All the processes were with a reaction temperature of 270 °C, a holding time of 30 min and an ethanol/water ratio of 1:1, based on previous studies (Wang et al., 2017) and preliminary experiments.

**Table 3:** Effect of catalyst types on elemental composition and element enrichment ratio in biocrude from catalytic liquefaction of MSS.

	CuSO_4_	ZnSO_4_	CoSO_4_	FeSO_4_	Blank
*Elemental composition (wt%, d)*					
Carbon	69.88	67.97	69.06	69.29	69.46
Hydrogen	9.82	9.57	9.64	9.17	9.36
Nitrogen	6.34	7.30	7.17	7.09	7.42
Sulfur	0.63	0.95	0.82	1.32	1.40
Oxygen^a^	13.33	12.49	10.92	13.13	9.03

*Element enrichment ratio (%)*					
Carbon	71.03	64.37	50.42	57.92	57.70
Hydrogen	68.02	61.76	47.96	52.24	52.99
Nitrogen	37.37	40.09	30.35	34.37	35.75
Sulfur	36.91	51.85	34.50	13.63	35.75
Oxygen	16.82	14.69	9.90	34.37	9.31

HHV (MJ/kg, d)	35.18	34.42	35.00	34.37	35.10

d: dry basis.

a Calculated by difference; Blank: without catalyst.

As shown in Fig. 1, biocrude with the highest yield (47.45%) was obtained with CuSO_4_ catalyst, compared with the biocrude yield without catalyst (38.78%). The higher liquefaction conversion was obtained in processes with ZnSO_4_ (92.68%) and CuSO_4_ (97.74%) catalysts. However, the ZnSO_4_ showed a less improvement than CuSO_4_ on biocrude yield, but this improvement verified that the zinc salt could improve the liquefaction process of woody feedstock in methanol or acetone(Aysu and Durak, 2016). Adding FeSO_4_ slightly improved the biocrude yield (from 38.78 to 39.02%) and decreased the liquefaction conversion (from 90.09 to 84.21%). This results was different from hydrothermal liquefaction of sludge (Malins et al., 2015). The CoSO_4_ catalyst showed a negative influence on both biocrude yield (34.08%) and liquefaction yield (86.29%). That was different from the catalytic liquefaction of microalgae *Nannochloropsis sp*., but in corresponding with the catalytic effect during the liquefaction of *D. tertiolecta* (Duan and Savage, 2011, Lin et al., 2017). These differences above might be explained by different feedstock characteristic and solvent environment. Further studies according to element analysis suggested that CuSO_4_ not only efficiently improved the biocrude yield but also changed the element composition of biocrude. The Sulfur content decreased by 55.0% (from 1.40% to 0.63%) while Nitrogen content decreased by 14.6 % (from 7.42% to 6.34%), and the major heteroatom contents in biocrude show a significant drop with CuSO_4_ catalyst. By adding CuSO_4_, although there were no significant improvements in element content of carbon or hydrogen, the main elements in petroleum-based fuel, the increase of element concentration ratio suggested that more carbon (from 57.70 to 71.03%) and hydrogen (from 52.99 to 68.02%) element concentrated into biocrude than those in biocrude without catalyst. Therefore, we chose CuSO_4_ as the best MSS liquefaction catalyst, which could improve biocrude yield, liquefaction conversion, and biocrude quality most by reducing the Nitrogen and Sulfur efficiently.

#### 3.1.2 Effect of copper salt with different anion type on liquefaction of MSS

Table 4 presents the effect of anion type of copper salt on liquefaction of MSS. Copper salts with nitrate, sulfate and chloridion anions were used as catalysts, respectively. Other operation conditions were all the same as those in Section 3.1.1. Obviously, the difference in anions of copper salts showed no significant influence on neither the biocrude yield nor the liquefaction conversion. Further element analysis suggested that different anion types hardly changed the element composition of biocrude from catalytic liquefaction of MSS in EWCS. Moreover, adding sulfate or nitrate could not transfer the sulfur and nitrogen in the anions of copper salt catalysts into biocrude. Therefore, this investigation could imply the further utilization of copper polluted sludge in catalytic liquefaction of MSS as both feedstock and catalyst.

**Table 4:** Element analysis of biocrude with different copper salts.

	CuSO4	CuCl2	Cu(NO3)2	Blank
Elemental composition (wt%, d)				
Carbon	69.88	69.78	69.83	69.46
Hydrogen	9.82	9.79	9.75	9.36
Nitrogen	6.34	6.41	6.41	7.42
Sulfur	0.63	0.64	0.58	1.40
Oxygena	13.33	13.38	13.43	9.03
Biocrude yield (%)	47.45 ± 1.5	47.38 ± 0.7	47.43 ± 0.3	38.78 ± 1.7
Liquefaction conversion (%)	97.74 ± 1.1	97.69 ± 1.2	97.71 ± 0.9	90.09 ± 0.4
HHV (MJ/kg, d)	35.18	35.11	35.06	35.10

d: dry basis; a: calculated by difference; Blank: without catalyst.

### 3.2 Effect of operating condition on liquefaction of MSS

This section reports the effect of reaction temperature, holding time and ethanol-water ratio on the biocrude yield, liquefaction conversion and biocrude element content from MSS liquefaction.

#### 3.2.1 Effect of reaction temperature on liquefaction of MSS

**Fig. 2:**
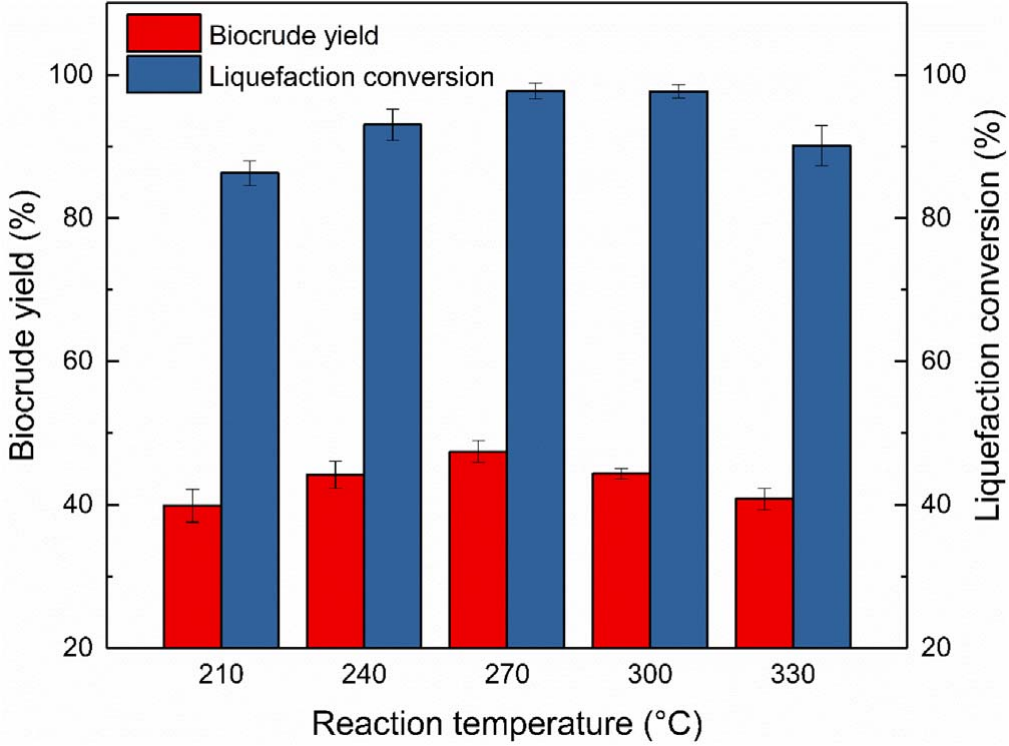
Effect of temperature on catalytic liquefaction of MSS.

In the present study, the reaction temperature provided the necessary heat for fragmentation of organic compounds and was believed to be the most important operating parameter (Ji et al., 2017, Ruiz et al., 2013). Fig. 2 reveals the effect of reaction temperature ranging from 210 to 330 °C on biocrude yield and liquefaction conversion from liquefaction of MSS. Other operating parameters were a holding time of 30 min, an E/W ratio of 1:1 and a CuSO_4_ dosage of 6 grams as the catalyst.

The lowest biocrude yield (39.88%) and liquefaction conversion (86.27%) was both acquired at the lowest temperature (210 °C). The two values gradually increased to 47.45% (biocrude yield) and 97.74% (liquefaction conversion) while the reaction temperature rose to 270 °C, which was in the temperature range for biochar production via hydrothermal conversion (Kruse et al., 2013). The un-liquefied solid residues could be the biochar, but the liquefaction conversion suggested that liquefaction conversion was almost finished. With temperature further increasing, the biocrude yield gradually decreased to 40.82% (330 °C) while the liquefaction conversion hardly changed from 270 to 310 °C and then decreased to 90.12% at 330 °C. According to the supercritical temperature of EWCS with an E/W ratio of 1:1 in Table 2, the decrease of liquefaction conversion might be due to the re-synthesis of solid organic residues from biocrude at supercritical EWCS condition.

During the liquefaction process, the general reaction pathways can be described as the following steps: (1) depolymerization of biomass macromolecules such as polysaccharides, proteins or lipids to monomers; (2) decomposition of monomers by cleavage, dehydration, decarboxylation and deamination to reactive intermediate; (3) decomposition of these intermediate by condensation, cyclization and polymerization to biocrude, aqueous phase product and solid organic product; (4) decomposition and decarboxylation of solid organic product, biocrude and aqueous phase to gas phase product(Hietala et al., 2016, Huang et al., 2011, Toor et al., 2011, Valdez and Savage, 2013). These organic reactions strongly depended on the interaction between solvents and the reactants. The higher the reaction temperatures are, the higher the reaction pressure will be in this closed liquefaction system. The rapid increasing reaction pressure will further increase the density of ethanol-water co-solvent (Chan et al., 2014, Mazaheri et al., 2010), and the higher solvent density can effectively penetrate into the biomolecule of biomass and enhance decomposition and extraction. However, with further reaction temperature increasing to supercritical conditions, the growth of solvent density causes cage effect to inhibit the C—C bonds breakage in biomass biochemical molecules so that the fragmentation ended (Kabyemela et al., 1998).On the other hand, biocrude might turn into gas phase product because the higher temperature could enhance the decarboxylation and gasification (Brand et al., 2013), which may promote large molecules to convert to smaller and more volatile ones and reduce the biocrude yield (Yang et al., 2016). Therefore, an appropriate high temperature can improve the biocrude yield and liquefaction conversion, but at a too high reaction temperature, a decrease in yield and conversion rate was observed. 270 °C was an appropriate temperature for catalytic liquefaction of MSS with CuSO_4_ catalyst.

#### 3.2.2 Effect of holding time on liquefaction of MSS

The effect of holding time on liquefaction of MSS process are presented in Fig. 4. Catalytic liquefaction experiments with different holding time, varying from 0 min to 8 h, were performed at a reaction temperature of 270 °C, an E/W ratio of 1:1 and CuSO_4_ as the catalyst.

**Fig. 3:**
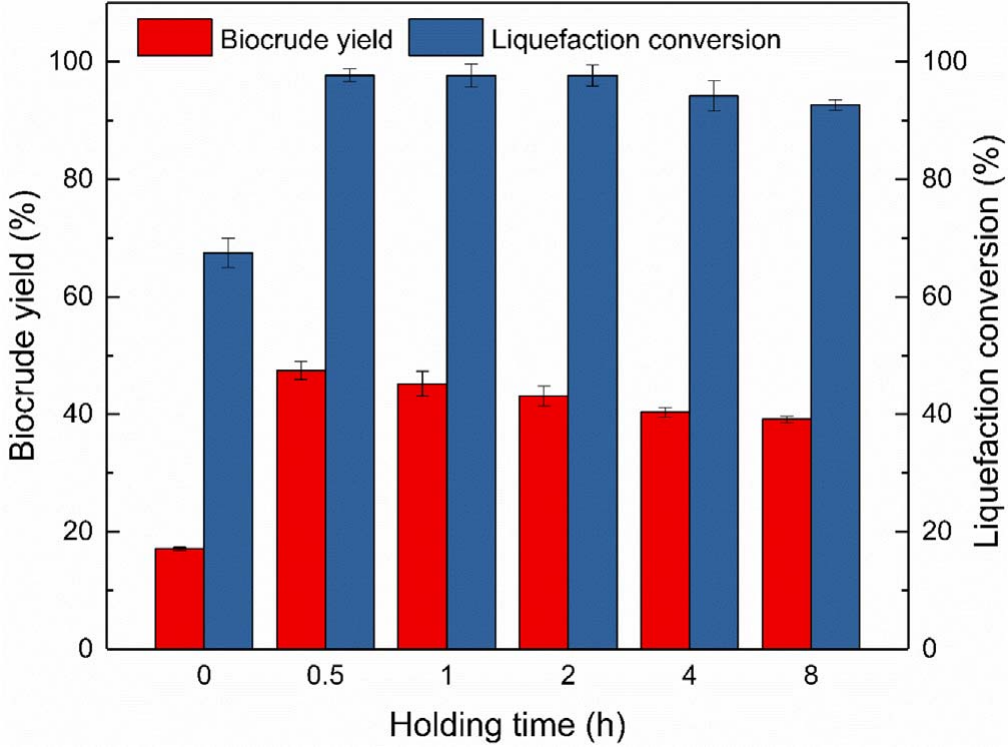
Effect of holding time on catalytic liquefaction of MSS.

As shown in Fig. 3, it is apparent that the biocrude yield and liquefaction conversion gradually increased when holding time prolonged from 0 to 30 min. In this time span, the holding time showed a noticeable impact on both biocrude yield and liquefaction conversion. The biocrude yield and liquefaction conversion, sharply rose from17.08% and 67.49% to 47.45% and 97.74%, respectively. In the liquefaction process, if the liquefaction system carried for enough time, the biomass would gain the opportunity for further cracking or re-polymerization reactions, which increased the final liquefaction conversion and biocrude yield (Malins et al., 2015). Obviously, at a holding time of 30 min, almost all the organic matters in the MSS have been decomposed and liquefied. When the holding time was in the range of 30 min to 2 h, the liquefaction conversion remained unchanged almost, until the holding time reached to 2 h. During this period, the biocrude yield steadily decreased. When the holding time gradually increased from 2 to 8 h, the liquefaction conversion decreased to 92.67% while the biocrude yield shrunk to 39.12% at 8 h. The reduction of biocrude yield with a longer holding time could be attributed to the further decomposition of biocrude into gases, aqueous, and solid phase products. The remained and further reduced of liquefaction conversion might be due to the further re-synthesis of organic solid residues from biocrude and other phase products (Valdez et al., 2014). Therefore, a suitable holding time, like 30 min, could convert the organic matters in MSS into biocrude phase product most efficiently. Shorter or longer holding time would not liquefy the organic matters in MSS entirely or misled our aim product biocrude into other phases. Further analysis to identify and differentiate the solid residues should be taken.

#### 3.2.3 Effect of E/W ratio on liquefaction of MSS

**Fig. 4:**
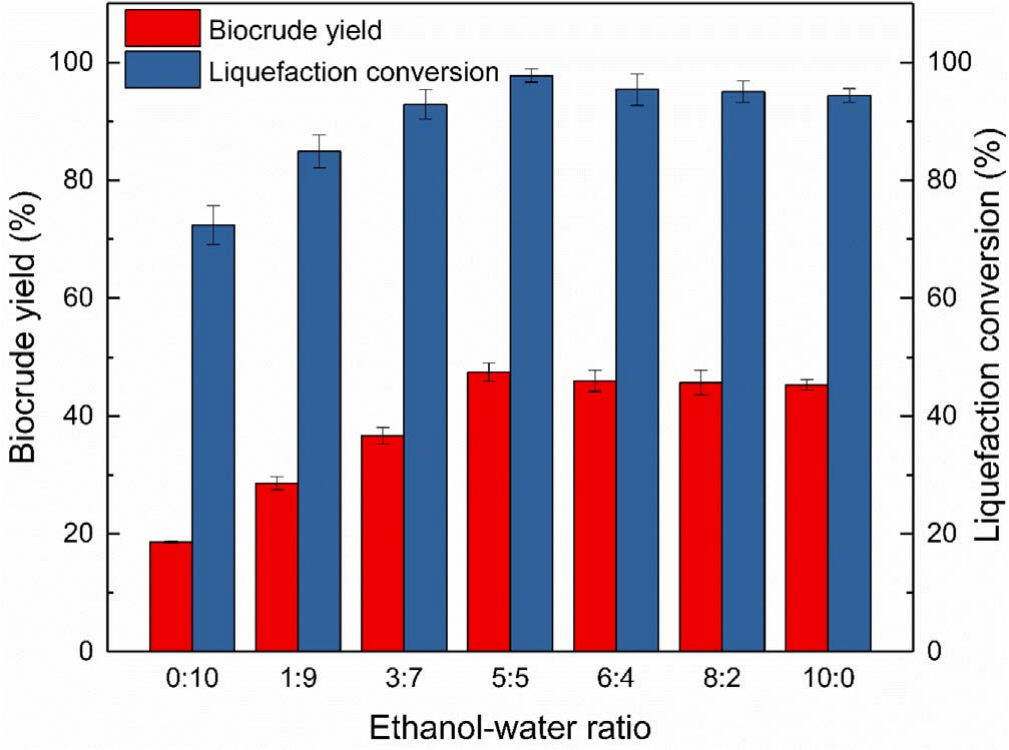
Effect of ethanol/water ratio on catalytic liquefaction of MSS with CuSO_4_.

MSS was liquefied in sub/supercritical Ethanol-water co-solvent with different volume ratio, varying from 0:10 to 10:0 (v/v) at 270 °C for 30 min with CuSO_4_ catalyst to determine the optimal ratio. As the reaction conditions presented in Table 2, all the liquefaction processes were operated in sub/super-critical conditions. The biocrude yield and the liquefaction conversion were plotted against the ethanol-water ratio as shown in Fig. 4.

Fig. 4 reveals the fact that neither pure ethanol nor water is as efficient as ethanol-water co-solvent for promoting liquefaction conversion or biocrude yield as the solvent medium. Ethanol-water co-solvent could provide synergistic effects on biocrude yield and liquefaction conversion of MSS, which was similar to the liquefaction process of microalgae *D. tertiolecta* in ethanol-water co-solvent (Chen et al., 2012). The liquefaction conversion (97.74%) and biocrude yield (47.45%) peaked with an ethanol-water ratio of 1:1. At this condition, the operating temperature (270 °C) was lower than the supercritical temperature of the ethanol-water solvent with an E/W ratio of 1:1 (313.97 °C) and suggested that the liquefaction process was carried in the subcritical environment. Compared with pure ethanol, pure water solvent showed a lower biocrude yield (18.63%) and liquefaction conversion (72.42%), this result corresponded to the literature (Huang et al., 2014). Therefore, to maximize the biocrude yield and liquefaction conversion, an E/W ratio of 1:1 used in this study was undoubtedly a better choice for a synergistic effect on MSS catalytic liquefaction.

### 3.3 Boiling point analysis

TGA, which shows the weight loss of biocrude vs. temperature in a constant flow of nitrogen, can be regarded as a miniature distillation process and measure the boiling point distribution of the biocrude (Zhu et al., 2015). Although some coking of biocrude or thermal degradation may occur at high temperature(Zhou et al., 2012), the TGA technique can provide a comparative analytical assessment of different condition and an estimate of boiling range of biocrude. The result of simulated distillation method is listed in Table 5. The biocrude was produced with a reaction temperature of 270 °C, a holding time of 30 min and an E/W ratio of 1:1.

**Table 5:** Boiling point distribution of the biocrude.

Name	Temperature range (°C)	Estimated boiling point range of biocrude (wt%, d)	
		With CuSO_4_	Without catalyst
Gasoline-line	30–170	16.06	12.09
Kerosene-like	170–250	16.42	14.41
Diesel-like	250–350	32.61	29.53
Fuel oil-like	350–500	22.27	28.48
Residue	500–800	1.52	2.24

d: dry basis.

As shown in Table 5, the fraction of the volatile biocrude that vaporized between 30 and 250 °C increased from 26.50 to 32.48 wt% by adding CuSO_4_ . Moreover, there were a certain amount of high-boiling-point compounds, which could not be identified by GC–MS, in biocrude samples. For two biocrude samples (obtained with/without CuSO_4_ catalyst), the light fraction with a boiling point <350 °C takes up 65.0 and 56.03 wt%, respectively. The distribution of biocrude samples indicates that most compounds in biocrude were light-weighted and had low boiling points. There were more light-weight compounds in biocrude by adding CuSO_4_ catalyst. By adding CuSO_4_ catalyst, the biocrude was more easily to pour from the evaporation flask after the rotary evaporation process. This change will be favorable for further separation and refining in industrial production of biocrude by HTL of MSS.

### 3.4 GC-MS results

The biocrude samples, which were obtained at 270 °C with a holding time of 30 min, an E/W ratio of 1:1 and CuSO_4_ as the catalyst, were analyzed by GC-MS to determine the main chemical components of biocrude further. During the analysis process, compounds with an identification probability more than 60% were taken into account. The percentage values, which were selected to represent the peak area proportion of every identified compound in biocrude by integration from the total ion chromatogram, were applied to indicate the proportions of individual compounds in biocrude indirectly. It should be noted that some low-molecule-weight components could get lost during the solvent evaporation processes for biocrude separation, while those high-boiling-point compounds could not elute from the GC column or might crack during the heating process in GC. Thus, only part of the components in biocrude can be characterized by GC-MS, and the percentage values do not present the actual concentration of each compound.

**Fig. 5:**
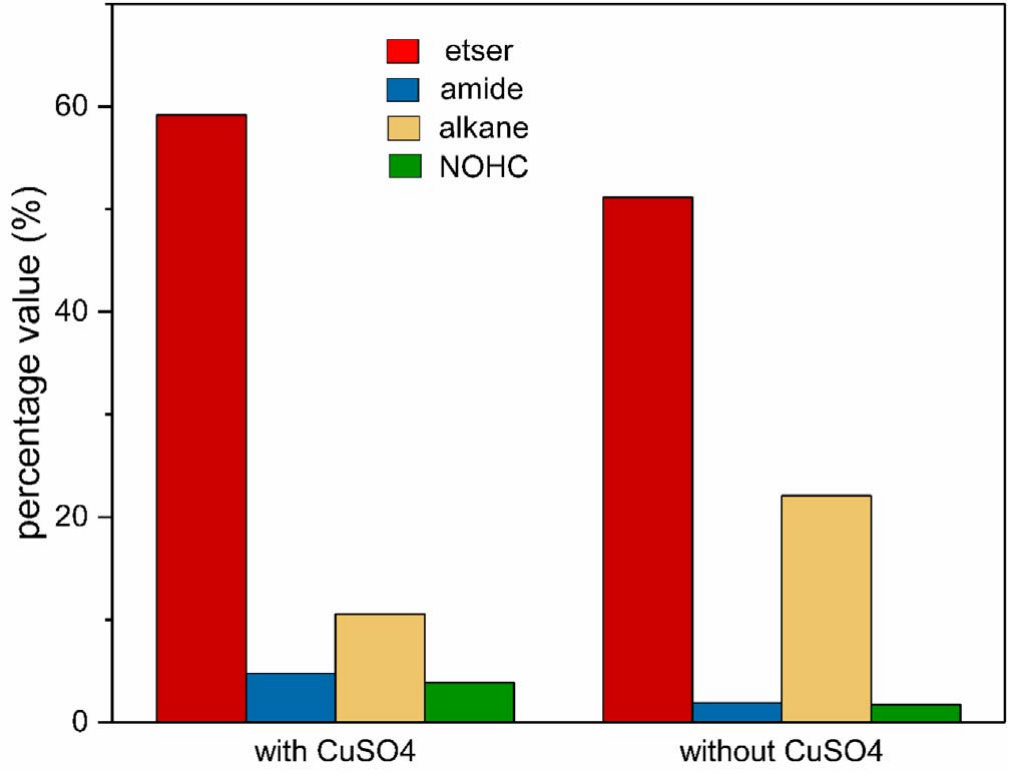
Classes distribution of major compounds in biocrude identified by GC-MS. NOHC: Nitrogen-and-Oxygen-containing heterocycle compounds.

Fig. 5 shows the change of quantified molecular components in the biocrude by adding the CuSO_4_ catalyst. To simplify the discussion, these identified compounds were apportioned into four different classes based on functionalities: esters, fatty acid amides, alkanes and Nitrogen-and-Oxygen-containing heterocycle compounds. As illustrated in Fig. 5, it indicated that esters (62.21 and 51.15% for conditions with/without CuSO_4_ catalyst, respectively) were the major chemical compositions of biocrude. For example, the percentage of ethyl palmitate increased from 27.25 to 31.84% by adding CuSO_4_. Based on the liquefaction environment and the products, there must be esterification reaction between ethanol and the fatty acid (palmitic acid) from hydrolysis of lipid (Changi et al., 2015, Zhou et al., 2012). The alkanes, especially the straight-chain alkanes or branched-chain alkanes, might come from the decarboxylation of fatty acid (Yeh et al., 2015). However, adding CuSO_4_ reduced the alkane content in biocrude, which was different from the catalytic deoxygenation of oxygen-containing biochemical molecular with Cu catalyst in super/sub-critical water (Ambursa et al., 2016, Loe et al., 2016). The fraction of nitrogen-containing compounds (amides and N-containing heterocycle compounds), which could be attributed to the protein in the MSS, increased from 3.65 to 8.36% but was still much less than that of esters and alkanes in the biocrude. Adding CuSO_4_ increased the content of amides from 1.92 to 4.75%. The amide products, as the reaction products between fatty acids and the amines produced by the cracking of amino acid (Teri et al., 2014), was detected and identified. A modicum of Nitrogen-and-Oxygen-containing heterocycle compounds was detected and might be synthesized in Maillard reaction between the hydrolysis products of protein and sugar (Zhang et al., 2016a). From the comparison in Fig. 5, adding CuSO_4_ could reduce the alkane content while increasing the amide and Nitrogen-and-Oxygen-containing compound contents as well as enhancing the synthesis of esters. An interesting discovery was the effect of CuSO_4_ on the competitive relation among reactions to product amides, esters and some of the alkanes, which might all come from the fatty acids. Adding CuSO_4_ could promote the production of esters most, then show a positive impact on the formation of amides and an impeditive effect on alkane production through the decarboxylation of fatty acid. However, the mechanism of catalysis is unknown and should be investigated in the future.

## 4 Conclusion

This study indicated that efficient conversion of MSS into biocrude in EWCS with CuSO_4_ catalyst via thermochemical liquefaction. The highest liquefaction conversion (97.74%) and biocrude yield (47.45%) were both obtained at 270 °C, 30 min, and an E/W ratio of 1:1. CuSO_4_ reduced the Nitrogen and Sulfur content in biocrude by 55.0% and 14.6%, respectively while increased the light oil content in biocrude. GC–MS identified that most compounds in biocrude were esters, alkanes, fatty acid amides, and Nitrogen-and-Oxygen-containing heterocycle compounds. MSS liquefied in EWCS with CuSO_4_ catalyst could be a potential way for converting MSS into fuel-like biocrude.
